# Marked CO_2_ Reduction to Generate C_1_–C_3_ Products Using Pt_0.9_Ru_0.1_/C-Based Membrane Electrode Assembly at Extremely Low Overpotentials

**DOI:** 10.1021/acsomega.4c10885

**Published:** 2025-03-03

**Authors:** Shofu Matsuda, Ryu Ishibashi, Minoru Umeda

**Affiliations:** † Department of Frontier Materials Chemistry, Graduate School of Science and Technology, Hirosaki University, 3 Bunkyo-cho, Hirosaki, Aomori 036-8561, Japan; ‡ Department of Materials Science and Technology, Graduate School of Engineering, Nagaoka University of Technology, 1603-1 Kamitomioka, Nagaoka, Niigata 940-2188, Japan

## Abstract

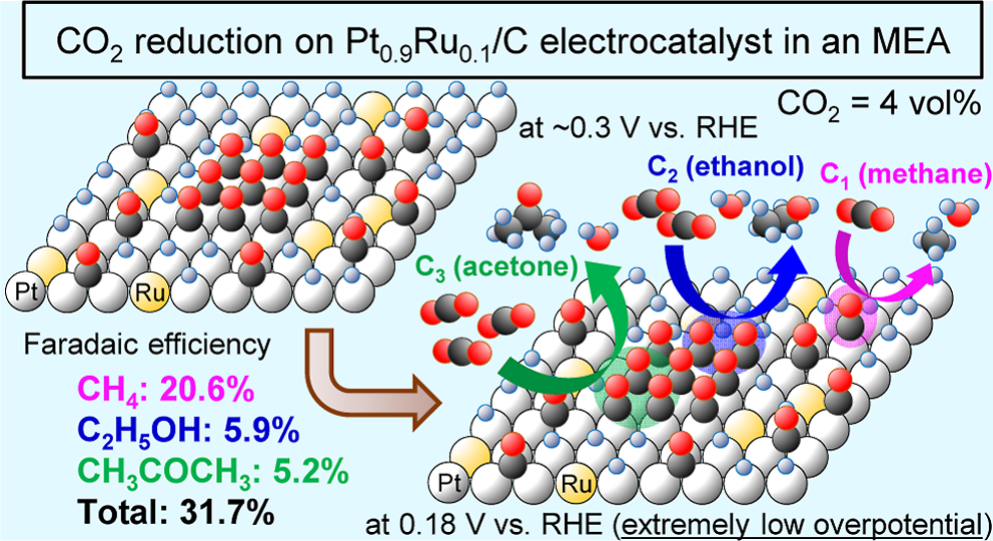

The electrochemical CO_2_ reduction reaction (CO_2_RR) over Pt electrocatalysts in an aqueous solution system yields mainly H_2_ and a slight amount of carbon-based products. This limitation can be overcome by using a membrane electrode assembly (MEA) containing a Pt/C electrocatalyst without any overpotential. However, the obtained CO_2_RR product was only CH_4_. In this study, we investigated the CO_2_RR using MEA containing a Pt_0.9_Ru_0.1_/C electrocatalyst. Consequently, not only methane as a C_1_ product but also ethanol as a C_2_ product and acetone as a C_3_ product were produced at extremely small overpotentials. This is the first time in the literature that ethanol and acetone were produced from the CO_2_RR over a Pt–Ru-based electrocatalyst. This fact was confirmed through mass spectrometry, gas chromatography, and isotope labeling experiments. The Faradaic efficiencies of CH_4_, C_2_H_5_OH, and CH_3_COCH_3_ were 20.6%, 5.9%, and 5.2%, respectively, and the total Faradaic efficiency was 31.7%. The three products were generated via the Langmuir–Hinshelwood mechanism involving adsorbed CO (CO_ads_) and H atoms on the electrocatalyst. The adsorption configuration of CO_ads_ determines the generation of methane, ethanol, and acetone. The C–C coupling reactions occurred through the formation of CO_ads_ clusters. Our findings promote the production of valuable C_2+_ compounds from the CO_2_RR, which is an important CO_2_ capture, utilization, and storage technology for realizing carbon neutrality.

## Introduction

The increasing global consumption of fossil fuels has led to an increase in the atmospheric CO_2_ levels, which contributes to global warming.^1^ CO_2_ can be converted to value-added chemicals and fuels via CO_2_ capture, utilization, and storage (CCUS) technologies;^2,3^ however, this method requires high energy because CO_2_ is an extremely stable molecule. The four major CCUS technologies involving thermochemistry,^4^ photochemistry,^5^ bioprocessing,^6^ and electrochemistry^7^ are promising, but have unresolved technical challenges. In this study, we explored the electrochemical CCUS technology because the electrochemical reduction of CO_2_ affords valuable compounds through a simple aqueous reaction at room temperature and atmospheric pressure.^8^

The CO_2_ reduction reaction (CO_2_RR) depends on the magnitude of the adsorption energy of CO adsorbed on an electrocatalyst (CO_ads_).^9−11^ Previous theoretical and experimental studies have reported the affinity of CO_ads_ for metal electrodes. CO_ads_ species can be easily desorbed from Au, Ag, and Zn electrocatalysts^12−18^ owing to their low adsorption energies. However, Pt, Ni, and Fe electrocatalysts did not or slightly yielded CO_2_RR products because the high binding energy between the metal and CO_ads_ prevents CO_ads_ desorption.^11,19−21^ Nevertheless, Cu electrocatalysts exhibit moderate adsorption energies with CO_ads_, yielding hydrocarbons and alcohols.^22−27^ Notably, C_2+_ products were obtained only from the CO_2_RR over Cu-based electrocatalysts.^7,28^ COO^–^_ads_ was the CO_2_RR intermediate for Pb and Sn electrocatalysts and was converted to HCOO^–^ and HCOOH via outer sphere electron transfer and protonation.^29−31^

Although the products of the CO_2_RR over Cu, Au, and Ag electrocatalysts demonstrate high Faradaic efficiency, they require very high overpotentials of ≥1 V.^32^ Using a nanoelectrocatalyst can lower the overpotential.^33−35^ However, the use of Cu nanoparticles in the CO_2_RR affords byproducts and lowers the Faradic efficiency of the target product. However, it is currently believed that Cu-based electrocatalysts are the most advantageous for the CO_2_RR.

Because the use of Pt electrocatalysts is expected to be a means of lowering the high overpotentials, many researchers have studied the CO_2_RR over Pt catalysts in the past. Giner reported that CO_2_ is electrochemically reduced at 0.00–0.25 V vs reversible hydrogen electrode (RHE) (without overpotentials) to generate “reduced CO_2_” over Pt.^36^ In situ infrared (IR) studies established this “reduced CO_2_” as CO_ads_.^37,38^ However, Pt electrocatalysts do not further reduce CO_ads_ because they are poisoned by CO_ads_.^39^ Hence, Pt has long been recognized as an inactive catalyst for CO_2_ reduction.

The employment of membrane electrode assembly (MEA)^40^ made a game change of CO_2_ reduction performances of Pt electrocatalysts. This MEA comprised a Nafion-based ion exchange membrane with Pt/C cathode and anode electrocatalysts and gas diffusion layers bonded to both sides. The MEA successfully converted CO_2_ to CH_4_ at extremely low overpotentials of ≤0.1 V,^41^ and no other product was obtained.

Controlling the CO_2_ concentration facilitates the production of CH_4_. Diluting CO_2_ to 4 vol % with Ar enabled the coexistence of the CO_ads_ and adsorbed H atoms (H_ads_) on the Pt surface in a molar ratio suitable for CH_4_ generation.^42,43^ According to the Langmuir–Hinshelwood (L–H) mechanism involving CO_ads_ and H_ads_, CO_2_ is successively reduced to CO_ads_ and CH_4_.^44^ The Faradaic efficiency was further improved by strategically controlling the electrode potential, amount of carbon support, and water content of the system.^45−48^ Notably, the Faradaic efficiency of CH_4_ generation from CO_2_RR over Pt_0.8_Ru_0.2_/C was higher than that over Pt/C.^49^ In Pt–Ru alloy catalysts, Ru affects the electronic state of CO_ads_ and weakens the Pt–CO bond.^39,50,51^ Hence, finely controlling the Ru content of the Pt–Ru/C electrocatalyst is expected to further improve the CO_2_RR performance.

Therefore, in this study, we investigated the characteristics of the CO_2_RR over the Pt_0.9_Ru_0.1_/C electrocatalyst to identify the most effective Pt–Ru/C electrocatalyst for the CO_2_RR. The employment of the Pt_0.9_Ru_0.1_/C electrocatalyst is expected to increase the efficiency of the CO_2_RR because it contains a lot of Pt, which is the active site for the CO_2_RR, and also contains Ru, which weakens the Pt–CO bond strength. Consequently, we obtained an amazing result that in addition to CH_4_, C_2_H_5_OH and CH_3_COCH_3_ were also obtained as products of CO_2_RR at extremely small overpotentials. This fact was confirmed through mass spectrometry (MS), gas chromatography, and isotope labeling experiments. This is a phenomenon very specific to Pt_0.9_Ru_0.1_/C that is not observed at Pt/C, Pt_0.8_Ru_0.2_/C, and Pt_0.5_Ru_0.5_/C electrocatalysts. Overall, this paper describes a brand new CCUS technology that CO_2_ is converted to value-added chemicals and fuels for realizing carbon neutrality.

## Materials and Methods

### Pt–Ru/C Electrocatalysts

Pt/C (with 46.2 wt % Pt; TEC10E50E), Pt_0.9_Ru_0.1_/C (with 41.2 wt % Pt and Ru), Pt_0.8_Ru_0.2_/C (with 42.5 wt % Pt and Ru), and Pt_0.5_Ru_0.5_/C (with 49.5 wt % Pt and Ru; TEC66E50) powders were provided by Tanaka Kikinzoku Kogyo K.K. (Tokyo, Japan). Their crystal structures were determined via powder X-ray diffraction (XRD) patterns recorded using a Smart Lab 3 kW (Rigaku, Tokyo, Japan) with CuKα1 radiation (1.5405 Å). The X-ray photoelectron spectroscopy (XPS) of all Pt–Ru/C electrocatalysts were recorded using a Nexsa spectrometer (Thermo Fisher Scientific K.K., Tokyo, Japan) equipped with an AlKα radiation source (1486.6 eV) to evaluate the electronic state of Pt–Ru.

### MEA Cell Fabrication

An MEA cell was prepared using a previously reported procedure.^42,49^ A Nafion 117 membrane (DuPont de Nemours, Wilmington, DE, USA) was used as the proton exchange membrane and was treated with 0.5 M H_2_O_2_ and 0.5 M H_2_SO_4_ solutions before use. Water-repellent carbon paper TGP-H-060 (Toray Industries, Tokyo, Japan) was used as a gas diffusion layer. A 1 mg_metal_ cm^–2^ Pt_0.9_Ru_0.1_/C electrocatalyst on carbon paper was used as the cathode, and a Pt/C catalyst was used as the anode. The apparent electrode area was 9 cm^2^. These two electrodes were hot-pressed onto both sides of the Nafion 117 membrane at 140 °C to afford an MEA. A RHE was prepared by placing the Pt/C electrocatalyst on the Nafion 117 membrane on the anode side. A single cell with a Pt_0.9_Ru_0.1_/C cathode was formed by sandwiching the fabricated MEA between gaskets, carbon separators with parallel flow paths, and stainless-steel plates. For comparison, single cells with Pt/C, Pt_0.8_Ru_0.2_/C, and Pt_0.5_Ru_0.5_/C cathodes were fabricated by the same procedure. The schematic of the experimental setup is shown in Figure S1.

### Electrochemical Measurements and Product Analysis

The single cell was connected to a polymer electrolyte fuel cell (PEFC)-operating apparatus (FCG-20S, ACE, Kanagawa, Japan), and the electrochemical measurements were performed by controlling the cathode potential (vs RHE) using a potentiostat (Vertex5A, Ivium Technologies B.V., Eindhoven, The Netherlands) at the cell temperature of 40 °C. All measurements were conducted by supplying fully humidified CO_2_/Ar mixed gas to the cathode and fully humidified H_2_ gas to the anode at flow rates of 50 mL min^–1^. Fully humidified H_2_ gas was supplied to the RHE at a flow rate of 10 mL min^–1^. The purities of CO_2_, Ar, and H_2_ gases were >99.995%, >99.998%, and >99.999%, respectively.

For cyclic voltammetry (CV), the cathode was scanned at a rate of 10 mV s^–1^ at the electrode potentials of 0.05–1.0 V vs RHE for Pt/C, 0.05–0.70 V vs RHE for Pt_0.9_Ru_0.1_/C and Pt_0.8_Ru_0.2_/C, and 0.08–0.70 V vs RHE for Pt_0.5_Ru_0.5_/C. For the potential-step experiment of Pt_0.9_Ru_0.1_/C, the cathode was stepped 14 levels in a negative direction every 2 min at the electrode potentials of 0.40–0.02 V vs RHE. In these measurements, we evaluated the current observed at the potential from the perspective of energy conversion. Discussing the energy conversion efficiency, which indicates how much the energy input to the cathode contributes to the CO_2_ conversion, is important to evaluate the possibility of industrial (engineering) applications. Hence, the energy conversion efficiency of the cathodic reaction without considering the overpotential of the anodic reaction was calculated using the following equation^32^
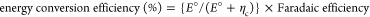
1where *E*^o^ and η_c_ represent the standard electrode potential of cathodic reaction and the cathodic overpotential, respectively.

During the electrochemical measurements at 4 vol % CO_2_, in-line MS was conducted at the ionization voltage of 23 eV by introducing the cathode exhaust gas directly into a mass spectrometer (JMS-Q1050GC, JEOL, Tokyo, Japan). The time lag for the in-line MS response was adjusted by H_2_ evolution detection (7 s). Calibration curves for quantitative evaluations were obtained using Ar-balance standard gases of 101 ppm of CH_4_, 108 ppm of C_2_H_5_OH, and 105 ppm of CH_3_COCH_3_.

## Results and Discussion

### Characterization of Pt–Ru/C Electrocatalysts

**Figure 1:**
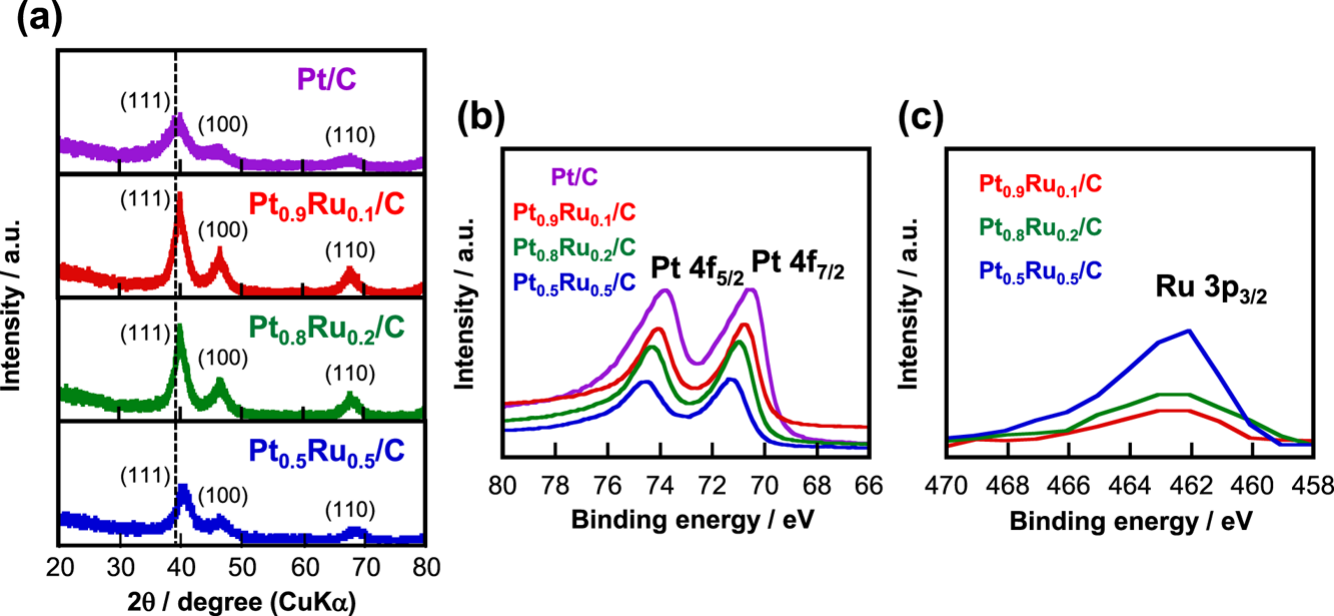
(a) XRD patterns and (b,c) XPS spectra of the Pt–Ru/C electrocatalysts.

Figure 1a shows the powder XRD patterns of the Pt/C, Pt_0.9_Ru_0.1_/C, Pt_0.8_Ru_0.2_/C, and Pt_0.5_Ru_0.5_/C electrocatalysts. All XRD patterns are nearly identical, indicating face-centered cubic lattice characteristics. However, the XRD peaks of the samples containing Ru were slightly shifted to angles higher than those of Pt/C (Figure S2a). Considering Bragg’s law and Vegard’s law, this result strongly supports the substitution of Pt with Ru, which has a smaller atomic radius than Pt, in the Pt lattice. In addition, the Ru-alone peaks corresponding to a hexagonal close-packed structure were not observed in the samples containing Ru (Figure 1a). These results are consistent with a previous report^52^ and indicate the successful formation of a Pt–Ru alloy. The Scherrer’s formula was applied to the (111) XRD peak of all the Pt–Ru/C electrocatalysts to calculate their crystallite diameter (∼3 nm; Figure S2b).

Figure 1b,c shows the Pt 4f and Ru 3p XPS spectra, respectively. Upon the addition of Ru to Pt, the Pt 4f peaks shifted to higher binding energies, whereas the Ru 3p peaks shifted to lower binding energies. These peak shifts are consistent with previous reports,^51,52^ indicating electron donation from Ru to Pt. Therefore, the Pt–Ru catalyst weakens the Pt–CO bond strength.^51^ In our previous study (in aqueous solution), it was also confirmed by the electrochemical measurement that the Pt–CO bond strength weakened when Ru was added.^39^

**Figure 2:**
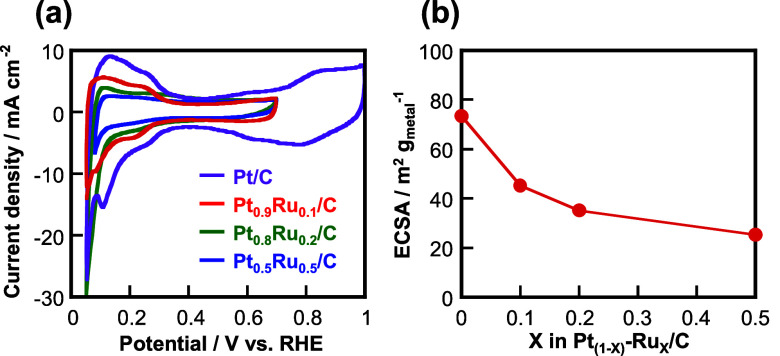
(a) Cyclic voltammograms of the Pt–Ru/C electrocatalysts at a scan rate of 10 mV s^–1^ in an Ar atmosphere and (b) Ru content dependence of the ECSA of the Pt–Ru/C electrocatalysts.

Figure 2a shows the cyclic voltammograms of Pt/C, Pt_0.9_Ru_0.1_/C, Pt_0.8_Ru_0.2_/C, and Pt_0.5_Ru_0.5_/C in a 100 vol % Ar atmosphere. Although the current decreased as the amount of Ru in Pt–Ru/C increased, the reduction and oxidation current peaks derived from H adsorption and H desorption, respectively, were observed at the potential range between 0.05–0.4 V vs RHE, which is consistent with our previous reports.^39,49^Figure 2b shows the electrochemical surface areas (ECSAs) of the Pt–Ru/C electrocatalysts estimated according to the hydrogen adsorption method.^39,49,53,54^ The ECSAs were estimated using the Faradaic charges of H adsorption and desorption in the voltammograms shown in Figure 2a, the ideal Faradaic charge of H adsorption at the Pt electrode (210 μC cm^–2^), and the following equation.
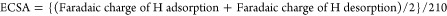
2

The ECSAs decreased as the Ru content increased, which suggests that the number of active sites of Pt–H (H_ads_) in the Pt–Ru/C electrocatalysts is dependent on the Ru content.

The XRD, XPS, and CV results revealed that Pt–Ru was certainly alloyed and no discerning characteristics of Pt_0.9_Ru_0.1_/C was observed among the four electrocatalysts.

### Comparison of CO_2_RR Performance of Pt–Ru/C Electrocatalysts

**Figure 3:**
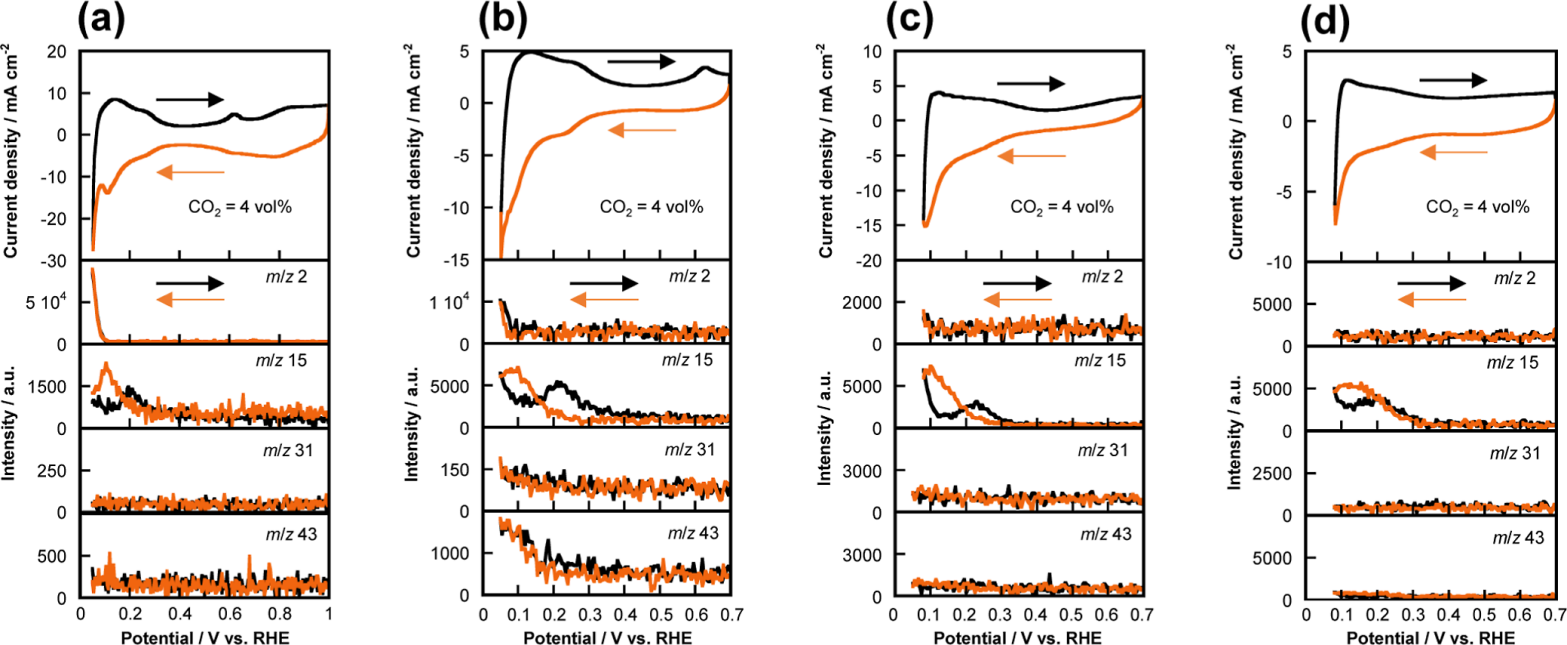
Cyclic voltammograms of the (a) Pt/C, (b) Pt_0.9_Ru_0.1_/C, (c) Pt_0.8_Ru_0.2_/C, and (d) Pt_0.5_Ru_0.5_/C electrocatalysts at 4 vol % CO_2_ in Ar gas and the associated potential dependence of the MS signals of *m*/*z* 2, 15, 31, and 43. Scan rate: 10 mV s^–1^.

Figure 3 shows the cyclic voltammograms of the different Pt–Ru/C electrocatalysts at 4 vol % CO_2_ in Ar gas and the corresponding MS signals at *m*/*z* 2, 15, 31, and 43. The use of 4 vol % CO_2_ allows the coexistence of CO_ads_ and H_ads_ on the electrocatalyst, thus affording CH_4_.^42−44^ As shown in cyclic voltammograms, the existence of large amount of H_ads_ is certain because the integrated oxidation current derived from H_ads_ desorption at the potential range of 0.05–0.4 V vs RHE is much larger than the integrated oxidation current derived from CO_ads_ desorption at the potential around 0.6 V vs RHE. Figure S3a shows the cyclic voltammograms of the Pt_0.9_Ru_0.1_/C electrocatalyst at various CO_2_ concentrations. We can observe that the oxidation current at the potential range of 0.08–0.4 V vs RHE decreases but the oxidation current between 0.4 and 0.7 V vs RHE increases as the CO_2_ concentration increases. This result indicates that CO_ads_ increases but H_ads_ decreases at higher CO_2_ concentrations. Both Faradaic charges estimated from Figure S3a are shown in Figure S3b. A trade-off relationship between the Faradaic charges for CO_ads_ (*Q*_CO_) and H_ads_ (*Q*_H_) was observed. Therefore, the ratio of CO_ads_ and H_ads_ on the Pt_0.9_Ru_0.1_/C electrocatalyst can be controlled by changing the CO_2_ concentration, which is also confirmed on the Pt/C and the Pt_0.8_Ru_0.2_/C electrocatalysts.^45,49^ Owing to the low product yield of the CO_2_RR products upon using the Pt–Ru/C electrocatalysts at 100 vol % CO_2_, we used 4 vol % CO_2_ in this study. The *m*/*z* 2 and 15 signals correspond to H_2_ and CH_4_ (CH_3_^+^), respectively (Figure 3).^22,42^ Only CH_4_ was detected as the product of the CO_2_RR over the Pt/C, Pt_0.8_Ru_0.2_/C, and Pt_0.5_Ru_0.5_/C electrocatalysts, which is consistent with our previous report.^44^ As the Ru content of the catalyst increased, lower levels of H_2_ (<0.1 V vs RHE) were produced. This confirms the dependence of the ECSA (amount of H_ads_) on the Ru content (Figure 2). Notably, the *m*/*z* 31 and 45 signals were detected along with the reduction current below ∼0.2 V vs RHE for the Pt_0.9_Ru_0.1_/C electrocatalyst. These may correspond to moieties other than CH_4_.

### CO_2_RR Products over Pt_0.9_Ru_0.1_/C

**Figure 4:**
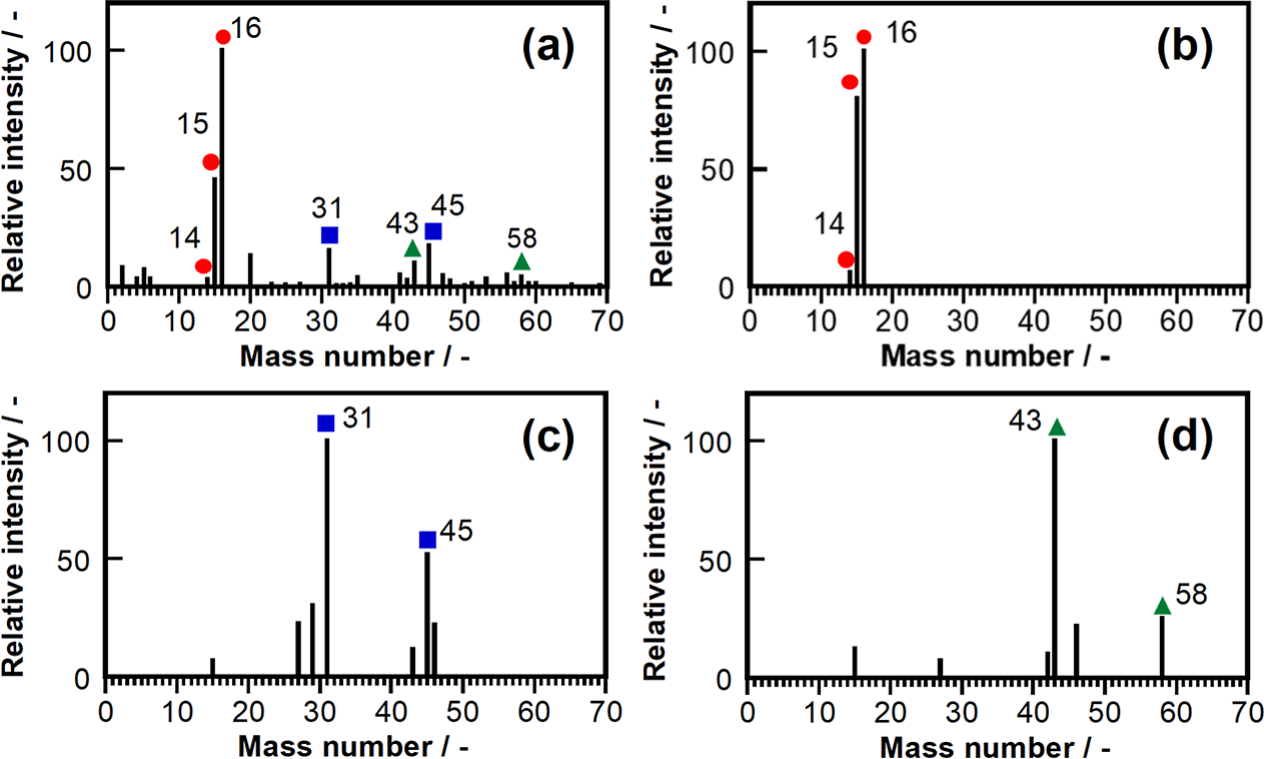
MS profiles of the (a) Pt_0.9_Ru_0.1_/C electrocatalyst at 0.1 V vs RHE during the negative scan of CV at 4 vol % CO_2_ and standard gases (b) 101 ppm methane, (c) 108 ppm ethanol, and (d) 105 ppm acetone.

The mass spectrum of the Pt_0.9_Ru_0.1_/C electrocatalyst at 0.1 V vs RHE (Figure 4) was compared with those of standard gases CH_4_, C_2_H_5_OH, and CH_3_COCH_3_. The *m*/*z* signals of 14, 15, and 16 (Figure 4a) were consistent with those of CH_4_ (Figure 4b). The *m*/*z* signals of 31 and 45 (Figure 4a) were consistent with those of C_2_H_5_OH (Figure 4c). The *m*/*z* signals of 43 and 58 (Figure 4a) were consistent with those of CH_3_COCH_3_ (Figure 4d). Hence, the CO_2_RR over Pt_0.9_Ru_0.1_/C generates ethanol and acetone in addition to methane.

We studied the CO_2_RR over the Pt_0.9_Ru_0.1_/C electrocatalyst via gas chromatography and isotope labeling experiments. The gas chromatogram confirmed methane, ethanol, and acetone as the CO_2_RR products (Figure S4). The isotope labeling experiment using ^13^CO_2_ gas revealed isotopically labeled methane, ethanol, and acetone (Figure S5). All three qualitative analyses confirm that methane, ethanol, and acetone are produced with high reproducibility from the CO_2_RR over the Pt_0.9_Ru_0.1_/C electrocatalyst.

**Figure 5:**
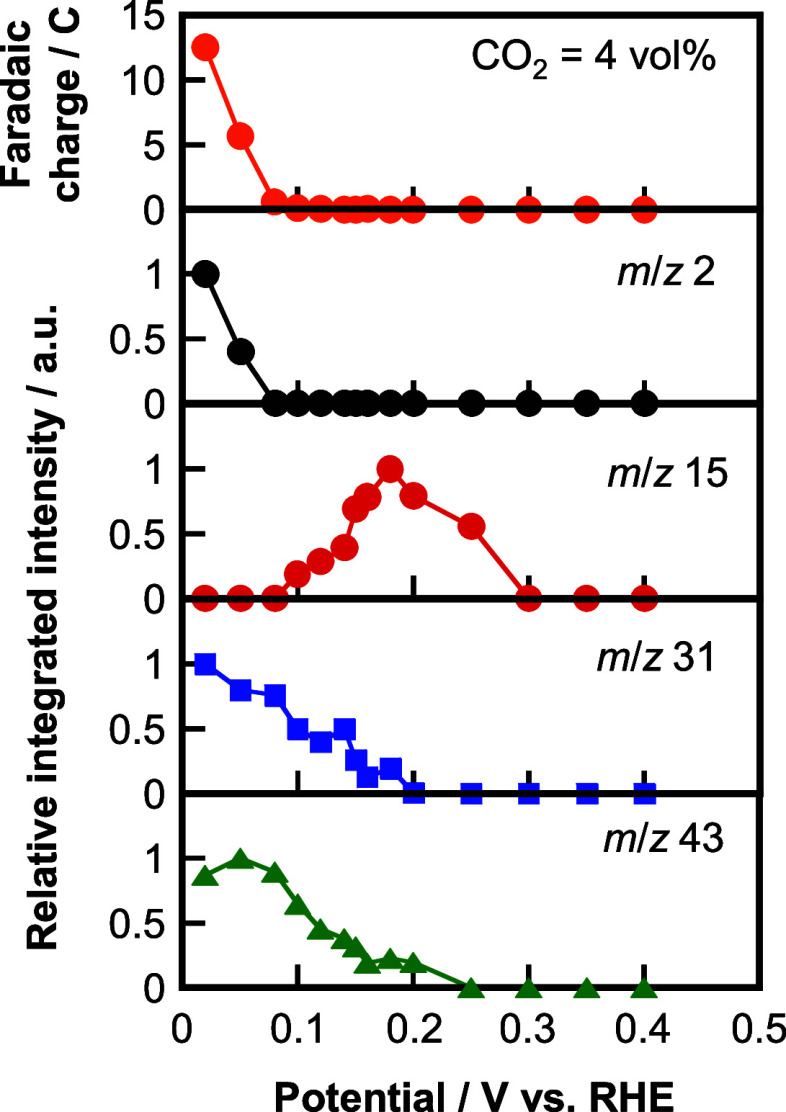
Potential dependence of Faradaic charges and integrated MS intensities of the *m*/*z* 2, 15, 31, and 43 signals during the CO_2_RR over the Pt_0.9_Ru_0.1_/C electrocatalyst at 4 vol % CO_2_ observed at each potential for 2 min. The potential was stepped 14 levels in a negative direction.

To evaluate the CO_2_RR at a steady state, we conducted stationary-potential CO_2_RR over the Pt_0.9_Ru_0.1_/C electrocatalyst. The potential was stepped 14 levels in the negative direction from 0.4 V vs RHE, while holding at each potential for 2 min. The reduction currents and MS signals observed between 1 and 2 min after holding at each potential were integrated to obtain the Faradaic charges and MS intensities, respectively. Figure 5 shows the potential dependence of the Faradaic charges and in-line MS intensities of the *m*/*z* signals of 2, 15, 31, and 43, corresponding to H_2_, CH_4_, C_2_H_5_OH, and CH_3_COCH_3_, respectively. Methane production began from ∼0.3 V vs RHE, reached a maximum at 0.18 V vs RHE, then decreased, and was no longer detected at 0.08 V vs RHE. Both ethanol and acetone appeared from ∼0.2 V vs RHE and were produced more at more negative potentials. The production of CH_4_, C_2_H_5_OH, and CH_3_COCH_3_ occur at different potentials (0.2–0.1 V vs RHE) from H_2_ evolution (≤0.08 V vs RHE). We compared the potentials at which methane, ethanol, and acetone were produced from the CO_2_RR over the Pt_0.9_Ru_0.1_/C electrocatalyst with their standard electrode potentials (*E*°).^55^
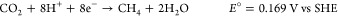
3
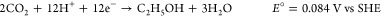
4
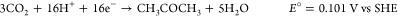
5

Remarkably, all products were generated at extremely low overpotentials.

The potentiostatic CO_2_RR at 4 vol % CO_2_ over the Pt_0.9_Ru_0.1_/C catalyst was conducted at 0.18 V vs RHE (hold for 300 s) stepped from 0.4 V vs RHE (Figure S6). Methane, ethanol, and acetone were produced continuously without H_2_ evolution. When the potential was then stepped to 0.03 V vs RHE from 0.18 V vs RHE, the methane production was observed at a high level but was quickly stopped because the H_ads_ required for the CH_4_ production are preferentially used for H_2_ evolution.^42−49^ However, ethanol and acetone production gradually decreased. Figure S7 shows the stationary-potential CO_2_RR over Pt_0.8_Ru_0.2_/C and Pt_0.5_Ru_0.5_/C electrocatalysts. Only methane was produced continuously at 0.2 V vs RHE, whereas ethanol and acetone were not detected.

**Figure 6:**
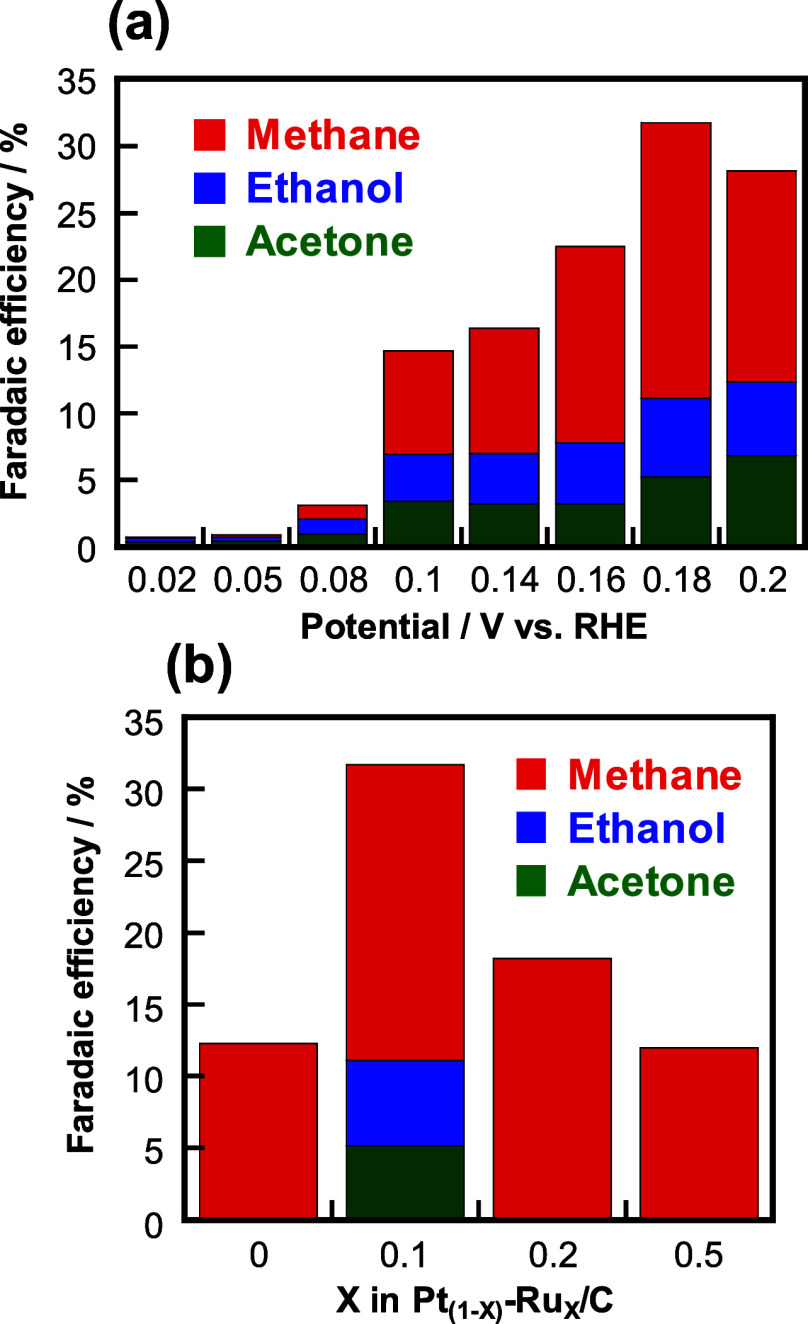
Faradaic efficiencies of methane, ethanol, and acetone achieved using a Pt_0.9_Ru_0.1_/C cathode at different (a) applied potentials (the data are taken from Figure 5) and (b) Pt–Ru/C compositions. The CO_2_ concentration and potential (vs RHE) were 4 vol % and 0.16 V for Pt/C, 4 vol % and 0.18 V for Pt_0.9_Ru_0.1_/C, 7 vol % and 0.20 V for Pt_0.8_Ru_0.2_/C, and 4 vol % and 0.22 V for Pt_0.5_Ru_0.5_/C, respectively.

Figure 6a shows the Faradaic efficiency of methane, ethanol, and acetone produced via the CO_2_RR over the Pt_0.9_Ru_0.1_/C electrocatalyst at different potentials. The Faradaic efficiency was estimated between 11–300 s after holding the potential to remove the influence of charging the electric double layer, which is a non-Faradaic reaction. The Faradaic efficiency of each product varied depending on the applied stationary electrode potential. In particular, we observed high Faradaic efficiencies of methane, ethanol, and acetone (20.6%, 5.9%, and 5.2%, respectively) at 0.18 V vs RHE. H_2_ evolution was observed only at 0.05 and 0.02 V vs RHE. Figure 6b compares the Faradaic efficiencies of the products obtained over the four Pt–Ru/C electrocatalysts. The data for Pt/C and Pt_0.8_Ru_0.2_/C were obtained from our previous reports.^45,49^ The highest Faradaic efficiencies of methane, ethanol, and acetone were achieved over Pt_0.9_Ru_0.1_/C among all catalysts. Moreover, the highest Faradaic efficiency of CH_4_ reported to date, as shown in Figure S8, and an even higher combined Faradaic efficiency of methane, ethanol, and acetone production (31.7%) were achieved over Pt_0.9_Ru_0.1_/C among all Pt–Ru-based electrocatalysts. Because H_2_ evolution was not observed at 0.18 V vs RHE, one possible process contributing approximately 70% of total Faradaic efficiency is the formation of CO_ads_ and H_ads_, which are not involved in the generation of the three products. The energy conversion efficiency of the CO_2_RR over the Pt_0.9_Ru_0.1_/C electrocatalyst was determined to be 31.7%, which is equivalent to that of CH_4_ and C_2_H_4_ production from CO_2_RR over the Cu-based electrocatalyst.^32^

In the future, it is expected to be important for increasing the product yield to control the Ru-content ratio of Pt–Ru alloy more precisely (e.g., employing a Pt_0.95_Ru_0.05_/C electrocatalyst), which will induce further increases in the Faradaic efficiency and the energy conversion efficiency.

We investigated the potential dependence of the amount of each product of the CO_2_RR over the Pt_0.9_Ru_0.1_/C electrocatalyst (Figure S9). The maximum amount of methane was produced at 0.18 V vs RHE, while the amounts of ethanol and acetone increased at more negative potentials. The maximum amounts of methane, ethanol, and acetone were 9.88 ppm (at 0.18 V vs RHE), 5.64 ppm (at 0.02 V vs RHE), and 5.02 ppm (at 0.02 V vs RHE), respectively, during a 1 min CO_2_ reduction. Nevertheless, the Faradaic efficiencies decreased as the potential became more negative (Figure 6a). This could be attributed to the large amount of H_2_ produced owing to the extremely high reduction current at potentials of <0.08 V vs RHE. Therefore, no correlation was observed between the Faradaic efficiency and the amount of each product because the former is affected by H_2_ evolution. As shown in Figure S10, the amounts of three products showed maximum at the CO_2_ concentration of 4 vol %.

In order to evaluate the durability of the Pt_0.9_Ru_0.1_/C electrocatalyst, the retention rates of the MS signals of *m*/*z* 15, 31, and 43 after 5 min potential holding at 0.18 V vs RHE were estimated from the data of Figure S6. As a result, the retention rates of *m*/*z* 15, 31, and 43 were determined to be as high as 90.6%, 94.6%, and 96.3%, respectively, which indicates the high stability of the CO_2_RR performance of the Pt_0.9_Ru_0.1_/C electrocatalyst.

### Mechanistic Analysis of CO_2_RR

Our experimental results revealed that the CO_2_RR over the Pt_0.9_Ru_0.1_/C electrocatalyst formed methane as a C_1_ product, ethanol as a C_2_ product, and acetone as a C_3_ product. The conversion of CO_2_ to value-added products involves a two-step reaction via the formation of intermediate CO_ads_.^9−11,28,44^ Hence, the adsorption configuration of CO_ads_ determines the final products.

To discuss the Pt–CO bond strength, the onset potentials of the generation of CH_4_ formed via CO_ads_ were determined from the potential dependence of the *m*/*z* 15 signal shown in Figure 3 for the four Pt–Ru/C electrocatalysts and plotted against the Ru content as shown in Figure S11. The onset potential shifted to more positive potentials as the Ru content increased. This indicates that the addition of Ru makes it easier for the CH_4_ production reaction to occur, i.e., weakens the Pt–CO bond strength.

**Figure 7:**
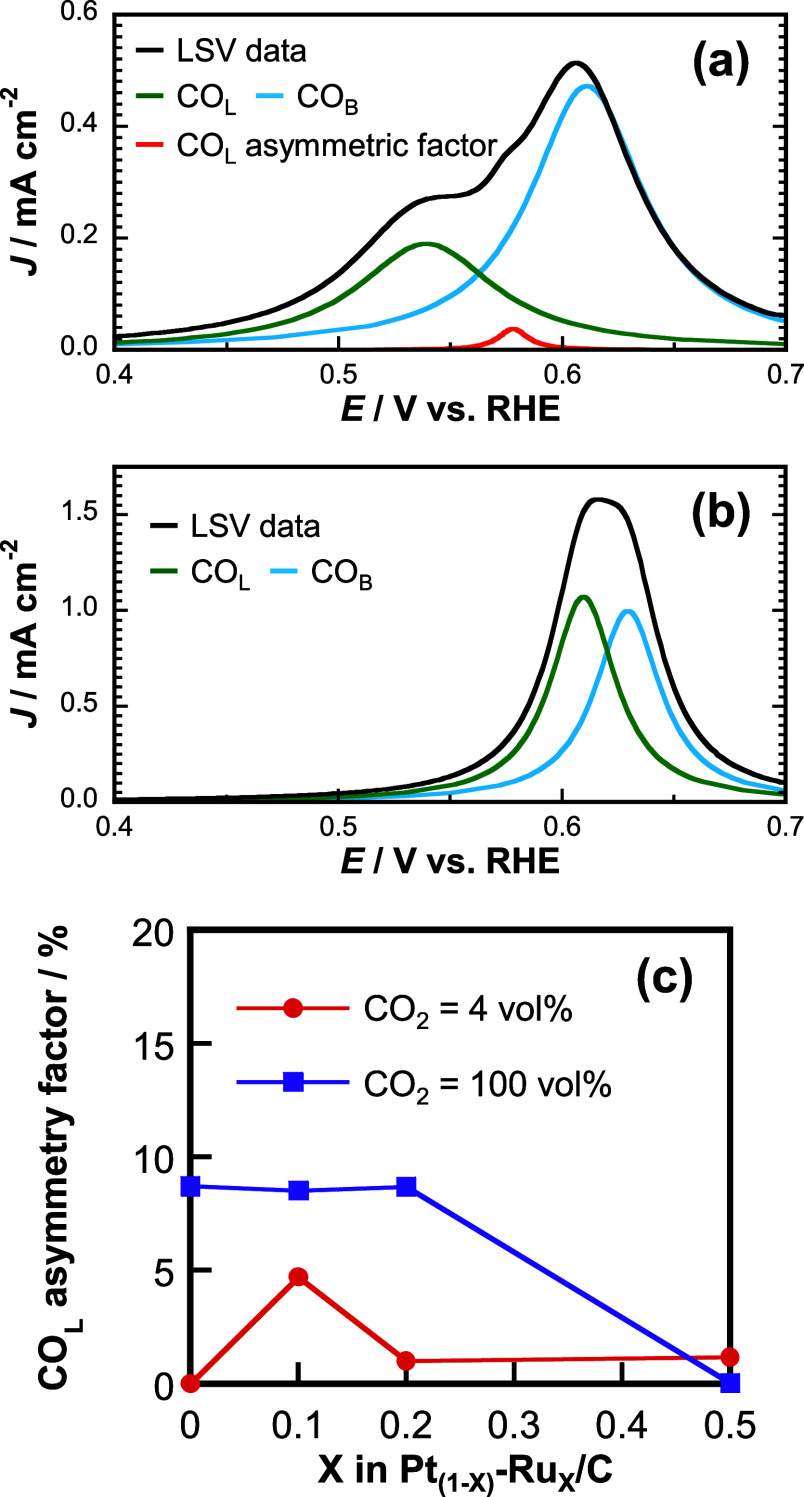
Anodic linear sweep voltammograms of the (a) Pt_0.9_Ru_0.1_/C and (b) Pt/C electrocatalysts at 4 vol % CO_2_. Curve fitting analysis was conducted to separate the peaks. *J* is the current density. (c) Ratio of the CO_L_ asymmetric factor to the total area of the separated peaks of different Pt–Ru/C electrocatalysts.

We conducted anodic linear sweep voltammetry (LSV) for the Pt_0.9_Ru_0.1_/C and Pt/C (black curves in Figure 7a,b) at a scan rate of 10 mV s^–1^ at 4 vol % CO_2_ after a potential sweep from 0.4 to 0.05 V vs RHE for CO_2_RR. The anodic current peak at ∼0.6 V vs RHE was attributed to the reoxidation of CO_ads_ formed by CO_2_RR.^39,48^ Two types of CO_ads_ were identified:^42,56^ CO_ads_ at the atop sites (CO_L_, linear CO observed at a lower oxidation potential) and that at hollow sites (CO_B_, bridge CO observed at a higher oxidation potential). The CO_ads_ species that are further reduced (converted to CH_4_) at a Pt electrocatalyst have been reported to be CO_L_, not CO_B_.^42^ Hence, it is important to deduce the adsorption configuration of CO_L_. The curve fitting analysis was performed using Origin 8.5 software assuming Lorentzian functions (Figure 7a,b). The assignment of the CO_L_ and CO_B_ peaks was performed with reference to LSV and IR data.^42^ The CO_L_ and CO_B_ peaks of Pt/C were completely separated (Figure 7b).^42^ However, the CO_L_ and CO_B_ peaks of Pt_0.9_Ru_0.1_/C could not be completely separated. Hence, we used a peak at ∼0.6 V vs RHE to fully separate the peaks (Figure 7a). This peak appears at a potential higher than that of the main CO_L_ peak. According to Figure S12, this asymmetric factor of CO_L_ could be attributed to the intermolecular interactions between the inner and outer CO_L_ of the CO_L_ aggregates (CO_L_ clusters) formed on the catalyst.^42^ We compared this LSV profile with those of the Pt_0.9_Ru_0.1_/C and Pt/C electrocatalysts obtained at 100 vol % CO_2_ (Figure S13).

Figure 7c shows the ratio of the CO_L_ asymmetric factor to the total area of the separated peaks obtained for the CO_2_RR over the four electrocatalysts at 4 and 100 vol % CO_2_. This ratio indicates the degree of CO_L_ clustering. Notably, the ratio at 4 vol % CO_2_ was high only for Pt_0.9_Ru_0.1_/C. In other words, the CO_L_ aggregates were formed on Pt_0.9_Ru_0.1_/C at ∼0.3 V vs RHE, while the CO_L_s were dispersed on Pt/C (Figure S14). Therefore, the C–C coupling of CO_L_s to yield ethanol and acetone products via the L–H mechanism was favorable on Pt_0.9_Ru_0.1_/C when the potential was stepped from the potential shown in Figure S14 to a potential where a subsequent reaction can occur.

**Figure 8:**
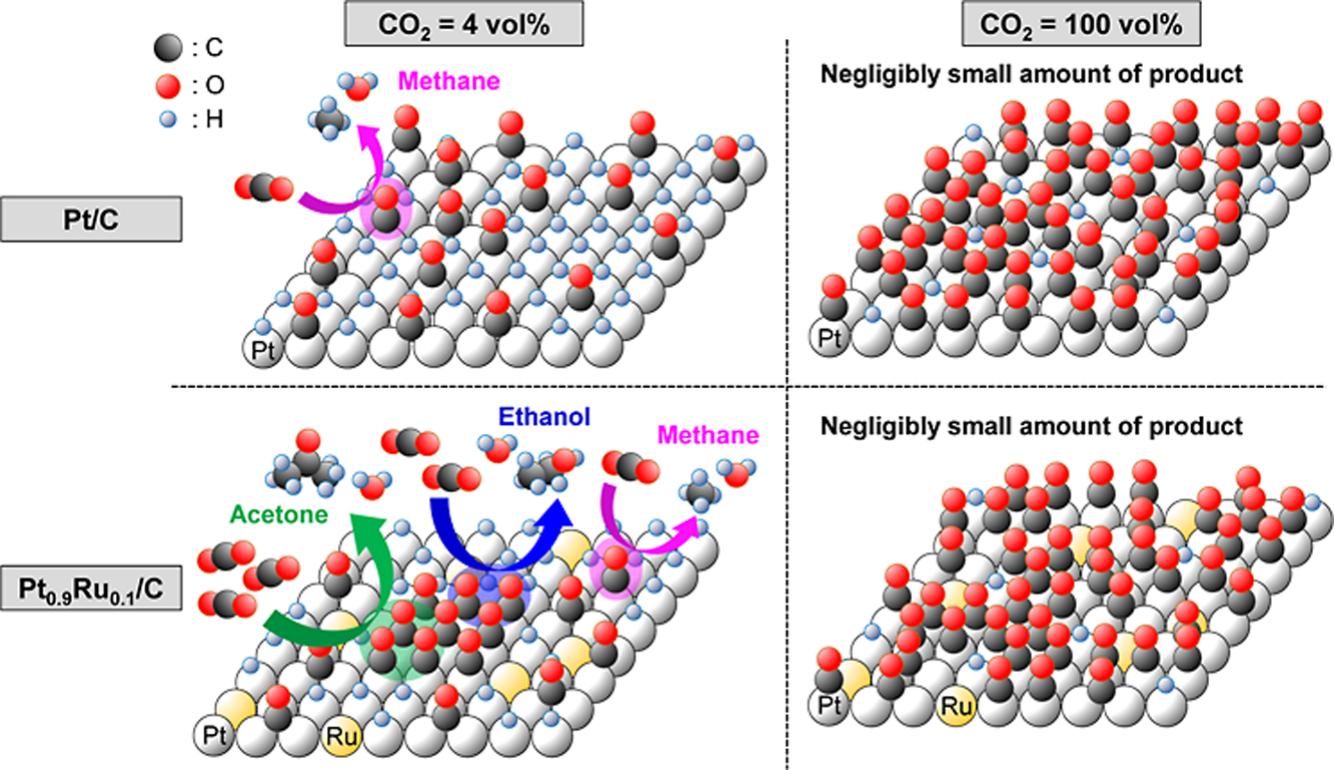
Schematic of the CO_2_RR at 0.18 V vs RHE (a condition under which a subsequent reaction occurs) over the Pt/C and Pt_0.9_Ru_0.1_/C electrocatalysts in 4 vol % and 100 vol % CO_2_ atmospheres. The negligibly small amount of product obtained at 100 vol % CO_2_ is attributed to the smaller number of H_ads_ than that of CO_ads_.

Based on our results, we proposed a plausible reaction mechanism for the CO_2_RR over the Pt_0.9_Ru_0.1_/C electrocatalyst (Figure 8). The schematic of the state in which the subsequent reaction occurs is shown in Figure 8. In the 4 vol % CO_2_ atmosphere, CO_2_ is reduced to the dispersed and aggregated CO_L_s on Pt/C and Pt_0.9_Ru_0.1_/C, respectively. The aggregated CO_L_s on Pt_0.9_Ru_0.1_/C have a higher probability of the C–C coupling reaction to form methane, ethanol, and acetone products via the L–H mechanism involving single and multiple CO_ads_ and H_ads_ (Figure 8).^28,44^ The coexisting CO_L_ clusters and H_ads_ species (Figure S14) generate the three products via specific dynamic reaction pathways (Figure 8) when the electrode potential is negatively stepped to 0.18 V vs RHE. Notably, only CH_4_ was produced over the Pt_0.8_Ru_0.2_/C and Pt_0.5_Ru_0.5_/C electrocatalysts owing to the formation of dispersed CO_L_s (Figure 7c). Moreover, a negligibly small amount of product was obtained over Pt–Ru/C at 100 vol % CO_2_ because of the extremely large aggregates of CO_ads_ and the absence of H_ads_ (Figure 8).

Finally, we compared the CO_2_RR performance of Pt_0.9_Ru_0.1_/C electrocatalyst with other recent studies (Cu-based electrocatalysts).^57−59^ As summarized in Table S1, the Faradaic efficiencies of the products are higher for Cu than for Pt_0.9_Ru_0.1_/C, but the high overpotential (∼1 V) is required for the Cu-based electrocatalysts. On the other hand, the products (CH_4_, C_2_H_5_OH, and CH_3_COCH_3_) are usually obtained with an extremely small overpotential when employing the Pt_0.9_Ru_0.1_/C electrocatalyst. This fact indicates that the Pt_0.9_Ru_0.1_/C electrocatalyst is an energetically advantageous electrocatalyst for the CO_2_RR.

## Conclusions

In this study, we evaluated the characteristics of the CO_2_RR over the Pt_0.9_Ru_0.1_/C electrocatalyst placed in a MEA cell. A 4 vol % CO_2_ feed was used to ensure the coexistence of the adsorbed CO (CO_ads_) and H (H_ads_) species on the Pt surface. The CO_2_RR produced CH_4_, C_2_H_5_OH, and CH_3_COCH_3_ at extremely low overpotentials (a stationary electrode potential of 0.18 V vs RHE) with the Faradaic efficiencies of 20.6%, 5.9%, and 5.2%, respectively, and a total Faradaic efficiency of 31.7%. To the best of our knowledge, these are the highest Faradaic efficiencies of the products achieved over Pt–Ru-based electrocatalysts. The characterization of Pt_0.9_Ru_0.1_/C showed a fully formed Pt–Ru alloy, and its cyclic voltammogram was nearly identical with that of Pt. However, electrochemical measurements revealed the role of Ru in forming clusters of linear CO_ads_ (CO_L_) on Pt_0.9_Ru_0.1_/C. Also, alloying Ru with Pt weakened the Pt–CO bond strength. These enable favorable C–C coupling reactions via the L–H mechanism to generate methane as a C_1_ product, ethanol as a C_2_ product, and acetone as a C_3_ product. Our findings promote the production of valuable C_2+_ compounds via the CO_2_RR, which is an important CO_2_ capture, utilization, and storage technology for realizing carbon neutrality.

## Supporting Information Available

Schematic of experimental setup; analysis of XRD patterns; CVs at various CO_2_ concentrations; gas chromatograms; MS profiles for ^13^CO_2_ reduction; stationary-potential CO_2_ reduction; amounts of products generated; CH_4_-generation onset potential; IR spectra of adsorbed CO; linear sweep voltammograms at 100 vol %; and schematic of CO_2_ reduction at ∼0.3 V vs RHE (PDF)
