# Supplementary material for: Marked CO2 Reduction to Generate C1–C3 Products Using Pt0.9Ru0.1/C-Based Membrane Electrode Assembly at Extremely Low Overpotentials
Source: ACS Omega. 2025 Mar 3;10(9):9630–8. doi: 10.1021/acsomega.4c10885 (PMC11904683; doi:10.1021/acsomega.4c10885)
Supplement: Supplementary file 1 — ao4c10885_si_001.pdf [file ao4c10885_si_001.pdf]

Supporting Information for

Marked CO_2 Reduction to Generate C_1–C_3 Products Using $Pt_{0.9}Ru_{0.1}$/C-based Membrane Electrode Assembly at Extremely Low Overpotentials

Shofu Matsuda,[†] *Ryu Ishibashi,*[‡] *Minoru Umeda**[,‡]

[†]Department of Frontier Materials Chemistry, Graduate School of Science and Technology, Hirosaki University, 3 Bunkyo-cho, Hirosaki, Aomori 036-8561, Japan

[‡]Department of Materials Science and Technology, Graduate School of Engineering, Nagaoka University of Technology, 1603-1 Kamitomioka, Nagaoka, Niigata 940-2188, Japan

*Corresponding author: mumeda@vos.nagaokaut.ac.jp

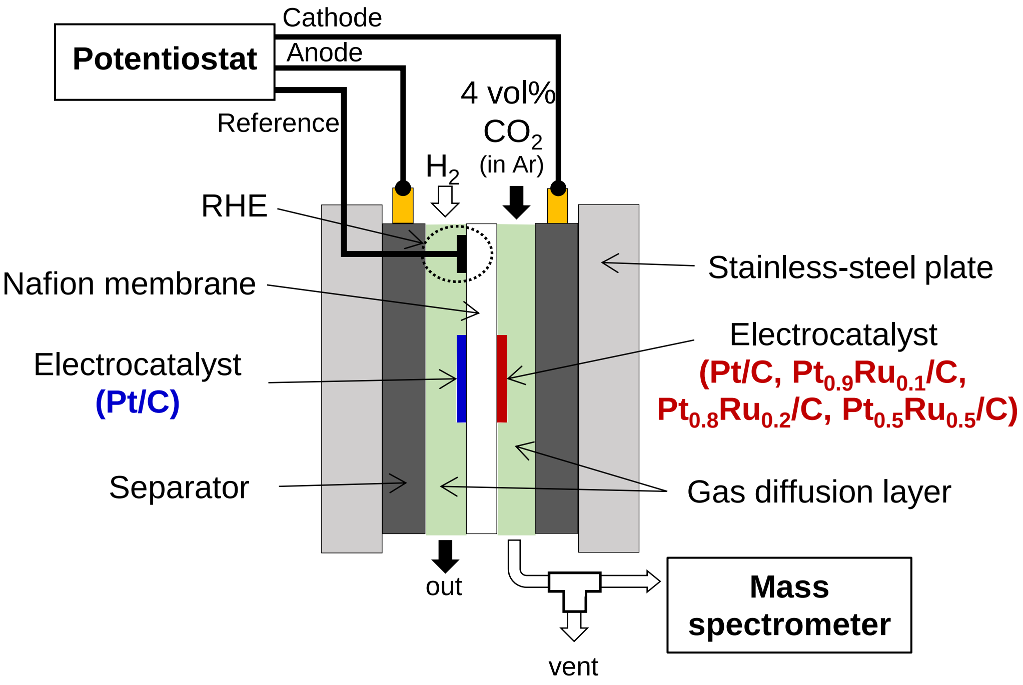

Figure S1. Schematic of the experimental setup in this study.

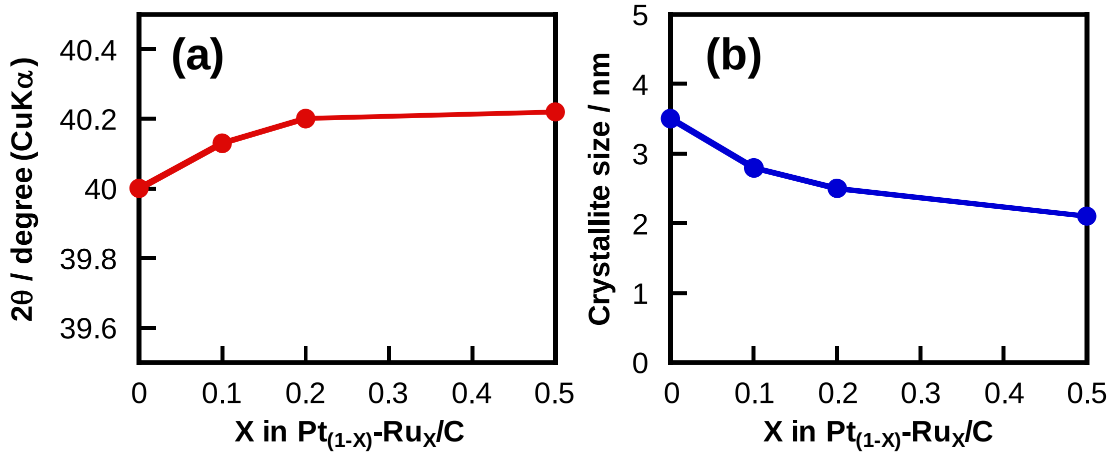

Figure S2. Analysis of XRD patterns of Pt-Ru/C shown in Figure 1a. The Ru-content dependence of the (a) (111) diffraction angle and (b) crystallite size calculated by applying Scherrer's formula to the (111) diffraction peak.

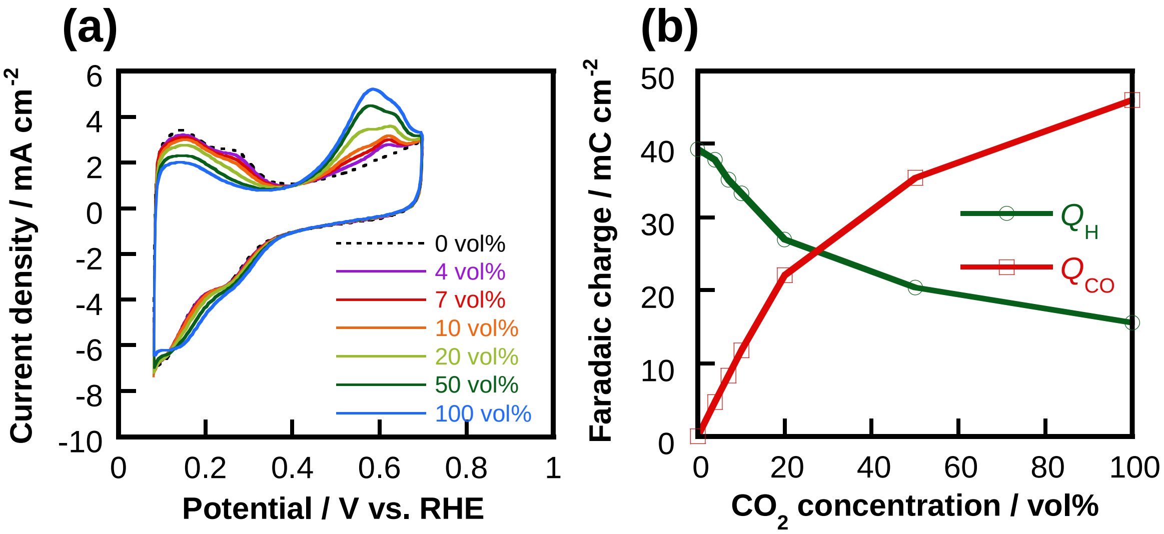

Figure S3. (a) Cyclic voltammograms of the $Pt_{0.9}Ru_{0.1}/C$ electrocatalyst at various CO_2 concentrations. (b) CO_2-concentration dependence of the faradaic charges of the oxidation-current peaks between 0.08–0.4 V vs. RHE (Q_H) and between 0.4–0.7 V vs. RHE (Q_{CO}) in the voltammograms shown in (a).

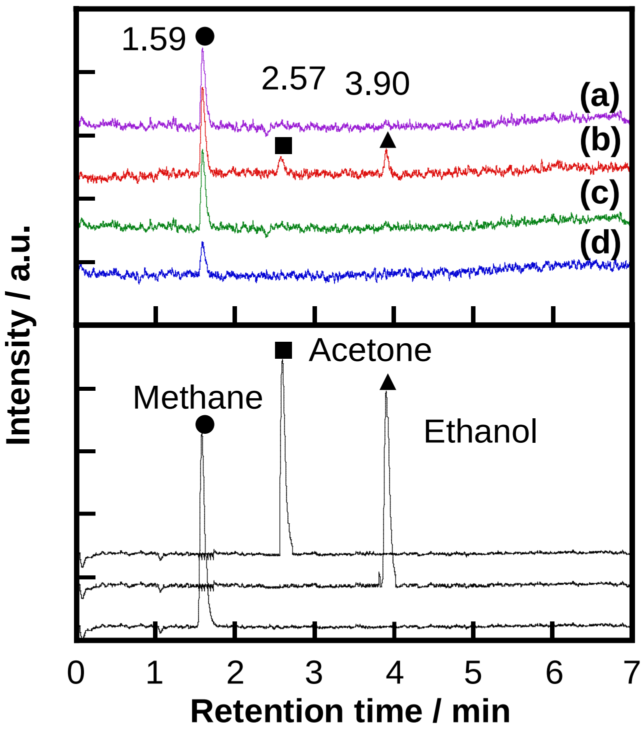

Figure S4. (Top) Gas chromatograms of the cathodic output gas from the (a) Pt/C cell at 0.16 V vs. RHE, (b) $Pt_{0.9}Ru_{0.1}$/C cell at 0.18 V vs. RHE, (c) $Pt_{0.8}Ru_{0.2}$/C cell at 0.20 V vs. RHE, and (d) $Pt_{0.5}Ru_{0.5}$/C cell at 0.20 V vs. RHE under a 4 vol% CO_2 atmosphere when holding the potential. (Bottom) Gas chromatograms of the standard gases of 101 ppm CH_4, 108 ppm C_2H_5OH, and 105 ppm CH_3COCH_3. A 6890 series gas chromatography system (Agilent) equipped with a flame ionization detector (FID) and a DB-WAX capillary column (Agilent) were used.

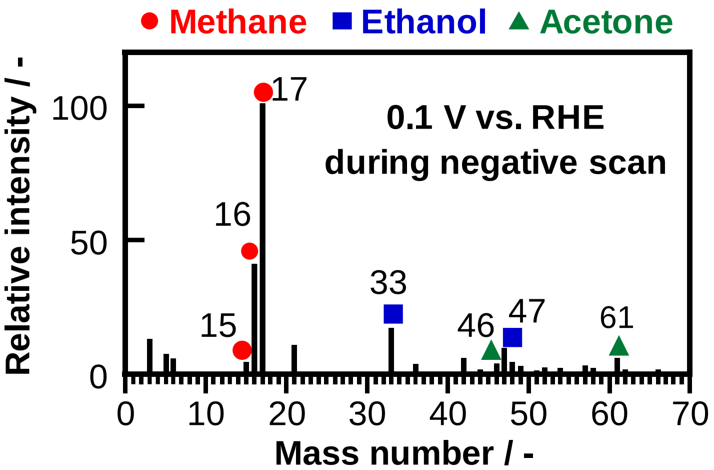

Figure S5. MS profiles of the $Pt_{0.9}Ru_{0.1}$/C electrocatalyst at 0.1 V vs. RHE during the negative scan of CV at 4 vol% $^{13}CO_2$ in Ar gas.

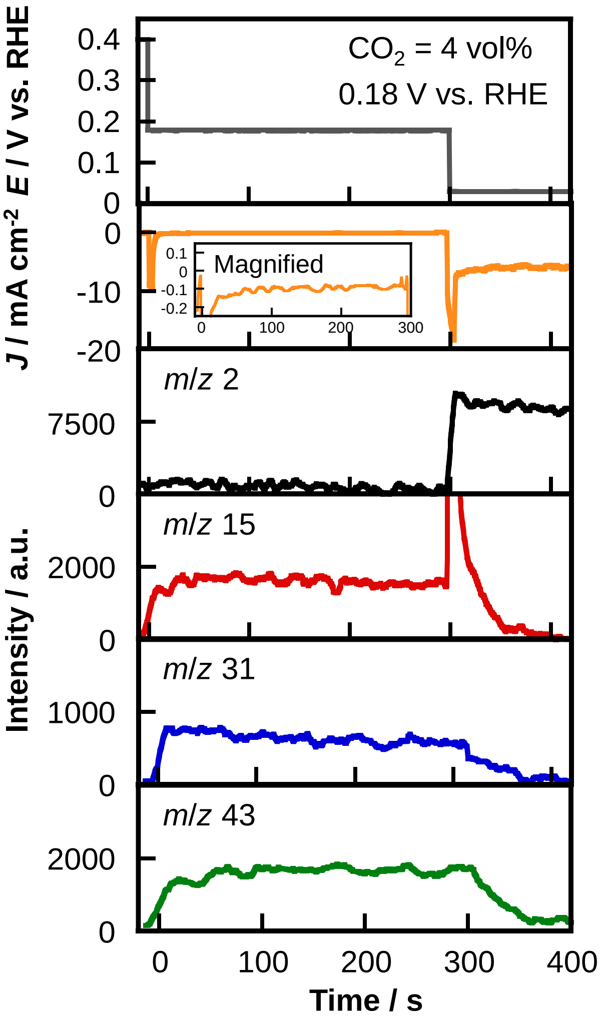

Figure S6. Potential program applied to the $Pt_{0.9}Ru_{0.1}/C$ cathode and the responses of current density (*J*) and in-line MS signals of *m*/*z* 2, 15, 31, and 43 in 4 vol% CO_2.

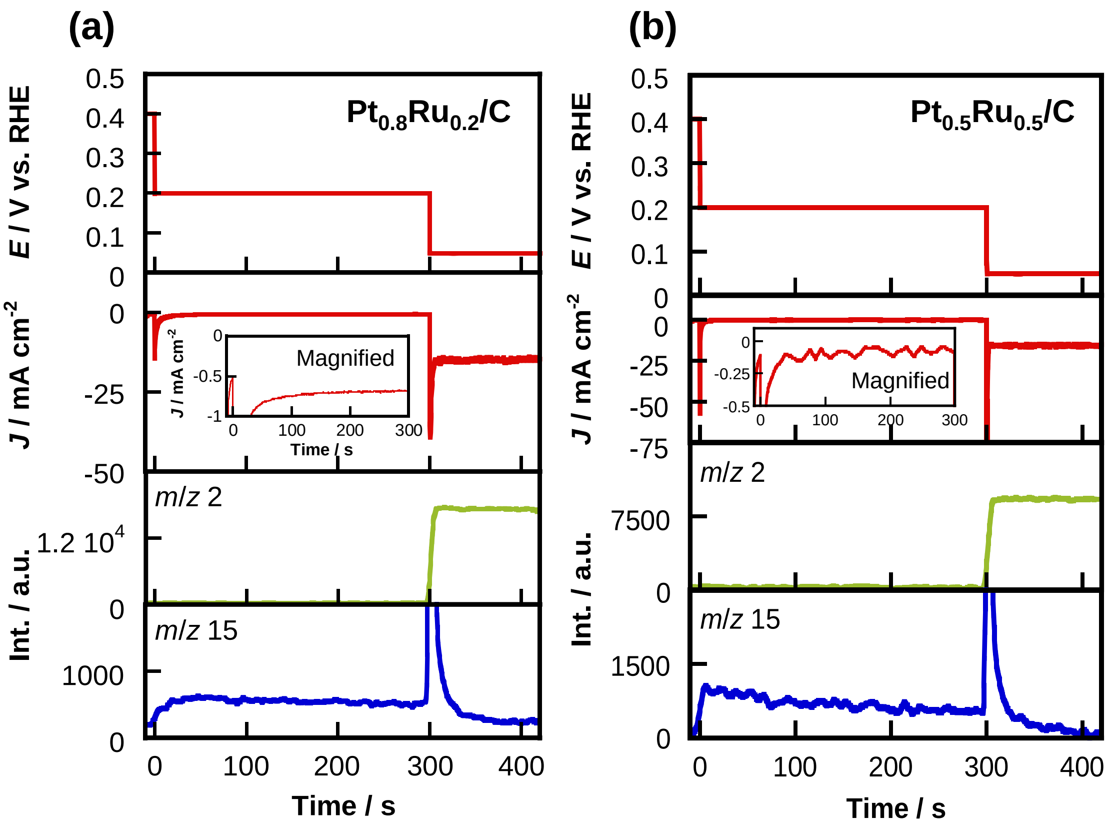

Figure S7. Potential programs applied to (a) the $Pt_{0.8}Ru_{0.2}/C$ electrocatalyst at 7 vol% CO_2 and (b) the $Pt_{0.5}Ru_{0.5}/C$ electrocatalyst at 4 vol% CO_2 and the responses of current density (*J*) and in-line MS signals of *m*/*z* 2 and 15. Panel (a) is reproduced with permission from ref 49 under a Creative Commons CC-BY license. Copyright 2021 Springer Nature.

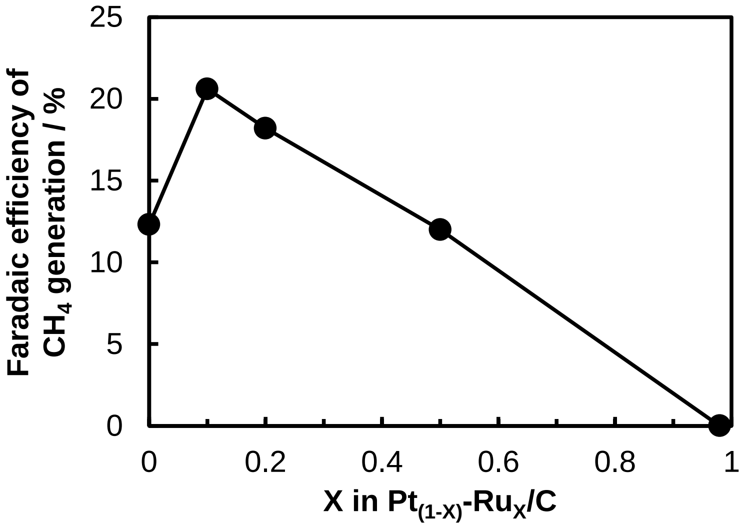

Figure S8. Faradaic efficiency of CH_4 generation from CO_2RR over the different Pt-Ru/C electrocatalysts.

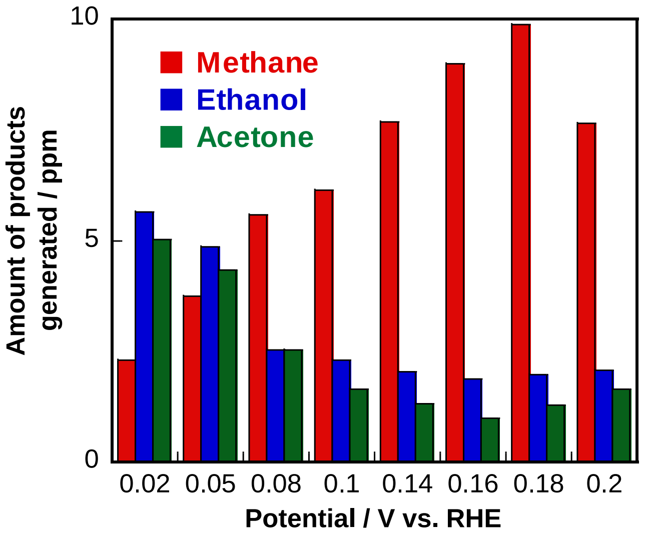

Figure S9. Amounts of methane, ethanol, and acetone produced by electrochemical CO_2 reduction over the $Pt_{0.9}Ru_{0.1}/C$ electrocatalyst in 4 vol% CO_2 at different electrode potentials. The data shown are from Figure 5.

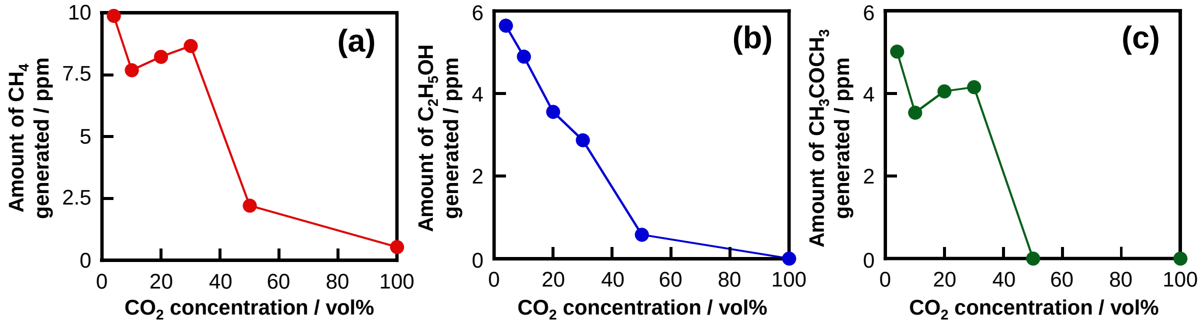

Figure S10. CO_2-concentration dependence of the amounts of (a) methane (at 0.18 V vs. RHE), (b) ethanol (at 0.02 V vs. RHE), and (c) acetone (at 0.02 V vs. RHE) produced by CO_2RR over the $Pt_{0.9}Ru_{0.1}/C$ electrocatalyst.

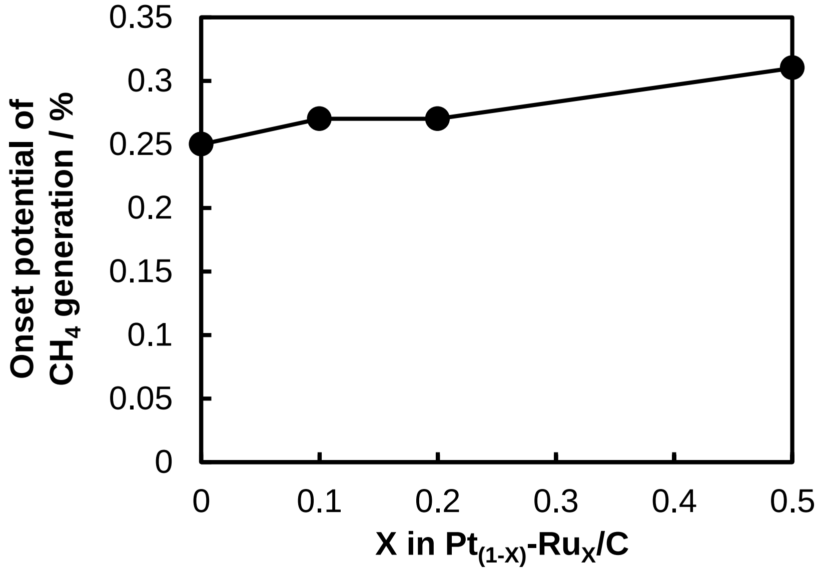

Figure S11. Dependence of the onset potential of CH_4 generation on the Ru-content in Pt-Ru/C electrocatalyst.

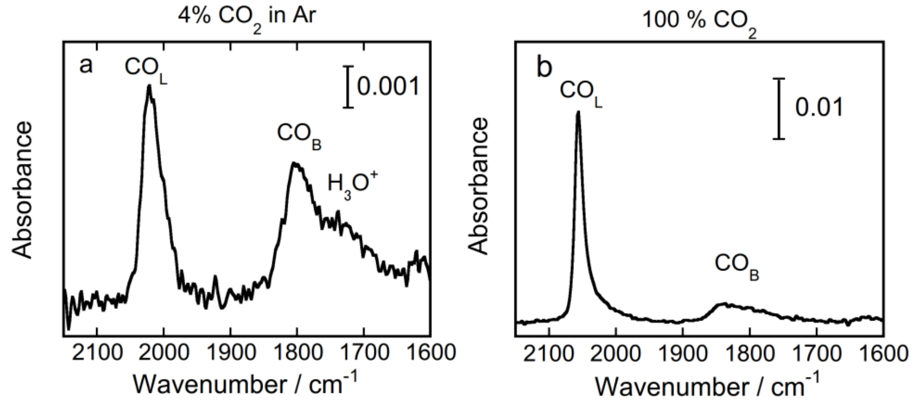

Figure S12. Surface-enhanced infrared absorption (SEIRA) spectra of CO adsorbed on a Pt thin film electrode in 0.5 M H_2SO_4 recorded at 0.2 V after bubbling the solution with (a) 4% CO_2-Ar mixture or (b) pure CO_2 for 5 min. Reproduced from ref 42. Copyright 2020 American Chemical Society.

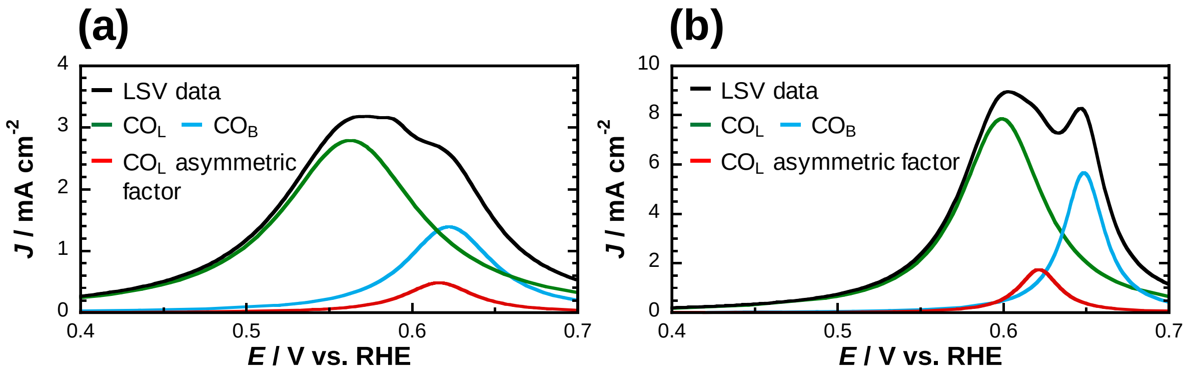

Figure S13. Anodic linear sweep voltammograms (black curve) of the (a) $Pt_{0.9}Ru_{0.1}$/C and (b) Pt/C electrocatalysts at 100 vol% CO_2 and the separated peaks after curve fitting analysis (green, blue, and red).

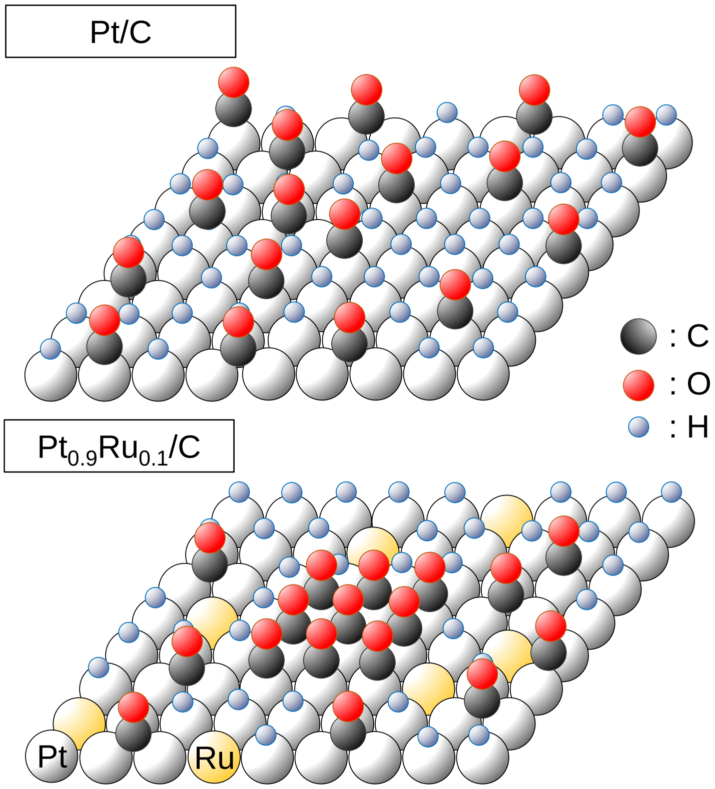

Figure S14. Schematic of the electrochemical CO_2 reduction at ~0.3 V vs. RHE (a condition under which a subsequent reaction does not occur) and at 4 vol% CO_2 over the Pt/C and $Pt_{0.9}Ru_{0.1}$/C electrocatalysts.

Table S1. Comparison of CO_2RR performance of $Pt_{0.9}Ru_{0.1}/C$ electrocatalyst with other recent studies (Cu-based electrocatalysts).

Electrocatalyst	Product	Overpotential / V	Faradaic efficiency / %	Ref
Cu-based	CH_4	1.63	73	57
	C_2H_5OH	1.18	51	58
	CH_3COCH_3	0.46-0.86	36.7	59
$Pt_{0.9}Ru_{0.1}/C$	CH_4	0.00	20.6	this study
	C_2H_5OH	0.0-0.06	5.9	
	CH_3COCH_3	0.0-0.08	5.2	
